# Within-host spatiotemporal dynamics of systemic *Salmonella* infection during and after antimicrobial treatment

**DOI:** 10.1093/jac/dkx294

**Published:** 2017-08-23

**Authors:** O Rossi, R Dybowski, D J Maskell, A J Grant, O Restif, P Mastroeni

**Affiliations:** University of Cambridge, Department of Veterinary Medicine, Cambridge, UK

## Abstract

**Objectives:**

We determined the interactions between efficacy of antibiotic treatment, pathogen growth rates and between-organ spread during systemic *Salmonella* infections.

**Methods:**

We infected mice with isogenic molecularly tagged subpopulations of either a fast-growing WT or a slow-growing Δ*aroC Salmonella* strain. We monitored viable bacterial numbers and fluctuations in the proportions of each bacterial subpopulation in spleen, liver, blood and mesenteric lymph nodes (MLNs) before, during and after the cessation of treatment with ampicillin and ciprofloxacin.

**Results:**

Both antimicrobials induced a reduction in viable bacterial numbers in the spleen, liver and blood. This reduction was biphasic in infections with fast-growing bacteria, with a rapid initial reduction followed by a phase of lower effect. Conversely, a slow and gradual reduction of the bacterial load was seen in infections with the slow-growing strain, indicating a positive correlation between bacterial net growth rates and the efficacy of ampicillin and ciprofloxacin. The viable numbers of either bacterial strain remained constant in MLNs throughout the treatment with a relapse of the infection with WT bacteria occurring after cessation of the treatment. The frequency of each tagged bacterial subpopulation was similar in the spleen and liver, but different from that of the MLNs before, during and after treatment.

**Conclusions:**

In *Salmonella* infections, bacterial growth rates correlate with treatment efficacy. MLNs are a site with a bacterial population structure different to those of the spleen and liver and where the total viable bacterial load remains largely unaffected by antimicrobials, but can resume growth after cessation of treatment.

## Introduction

Bacterial diseases pose a grave threat for humankind, causing approximately six million deaths per year.^1^ Antibiotics are key weapons against bacterial infections. However, antimicrobial treatment does not always result in the complete resolution of acute bacterial infections even when the pathogen retains susceptibility to the drugs used.^2^^,^^3^ Post-treatment persistence of antibiotic-susceptible bacteria is increasingly being recognized as a situation of great medical importance and can lead to disease reservoirs, continued transmission and in some cases within-host relapses, especially in immune-deficient individuals.^2^^,^^4–9^ The reasons why many antimicrobials are far less effective *in vivo* than in *in vitro* are difficult to explain. It is likely that key parameters of *in vivo* pathogen behaviour, such as location, division and spread within and between different organs, would have an impact on the therapeutic potential offered by antimicrobials.^10–18^

The aim of this study was to determine whether the *in vivo* net growth rate of *Salmonella* affects the efficacy of ampicillin and ciprofloxacin, representatives of two classes of antimicrobials (β-lactams and fluoroquinolones) commonly used for the treatment of systemic salmonelloses in mammalian species.^13^ We used a tractable murine *in vivo* model that captures many essential traits of systemic *Salmonella* infections of humans and other animals. This model captures, with the highest level of concordance, the pathogenesis of invasive salmonelloses in humans and other animals and has indicated the path for antimicrobial treatment.^8^^,^^9^^,^^19–23^ Murine infections with fast-growing bacteria mimic closely the pathogenesis of non-typhoidal salmonelloses while infections with slow-growing bacteria closely resemble typhoid and paratyphoid fever.

We compared ampicillin and ciprofloxacin because they exhibit differences in their mechanisms of action that can be predicted to result in different levels of dependence on bacterial division rates for optimal efficacy. Ampicillin treatment inhibits cell wall biosynthesis and therefore its bactericidal action depends on bacterial division. Ciprofloxacin inhibits DNA gyrase and DNA topoisomerase IV; these enzymes are involved in many cellular processes that involve duplex DNA (i.e. replication, recombination and transcription) and therefore the action of ciprofloxacin can be predicted to be less dependent on the division rate of the bacterial strain.^24^

Understanding the efficacy of antimicrobials in different organs is necessary to improve their *in vivo* use and to maximize treatment efficacy. We therefore asked whether the antimicrobials are equally effective in different tissues [i.e. spleen, liver, mesenteric lymph nodes (MLNs) and blood] and how bacterial spread of the infection between body sites is affected by the antimicrobial treatment and its cessation.

We infected mice intravenously with mixes of isogenic tagged strains (ITS) that contain specific nucleotide sequences that do not alter pathogen fitness, but enable the quantification of each tagged subpopulation *in vivo*.^12^^,^^25^ Using this approach we have already determined the dynamics of growth and spread of *Salmonella* within and between organs,^12^ and we have been able to understand how immune pressure and vaccines affect the infection process at the level of individual bacterial subpopulations.^26^

In this study, we compared the effect of antimicrobials on fast-growing wild-type ITS (WITS) and on slow-growing Δ*aroC* mutant ITS (MITS).^27^ We determined the total bacterial viable counts in spleens, livers, MLNs and blood before, during and after cessation of antimicrobial treatment. We also determined the presence and proportional frequency of each ITS within each organ as a proxy of the bacterial spread between organs or confinement of ITS to an individual body site.

## Materials and methods

### Bacterial strains

We generated eight fast-growing WITS^12^ and eight slow-growing Δ*aroC* MITS^27^^,^^28^ using the virulent *Salmonella enterica* serovar Typhimurium strain SL1344^27^ as the parent strain (details of the *aroC* defined mutation, and of MITS and WITS generation are listed in the Supplementary methods, Appendix 1 and Table S1, available as Supplementary data at *JAC* Online). Standard methods and reagents were used for molecular cloning^29^ and for integration of linear DNA fragments into the chromosome of *Salmonella* recipient cells using a modification of the method of Datsenko and Wanner.^12^^,^^30^ Each of the eight WITS or MITS contains a different 40 bp DNA signature tag in the same non-coding region of the chromosome; this enables the discrimination and quantification of each WITS- or MITS-tagged strain (subpopulation) in a mixed sample.^12^ Similar MICs of ampicillin and ciprofloxacin and similar net growth rates *in vitro* and *in vivo* were observed within each set of eight WITS and within each set of eight MITS (data not shown).

### Infections and antimicrobial treatments

Female, age-matched C57BL/6 mice were purchased from Envigo UK, and used when over 8 weeks of age (mean weight 20 ± 3 g). The mice were housed in specific pathogen-free containment facilities and were allowed water and food *ad libitum*. WITS and MITS were grown individually from glycerol stocks for 24 h at 37 °C on LB agar supplemented with the appropriate antimicrobials (50 mg/L kanamycin for WITS, 50 mg/L kanamycin and 20 mg/L tetracycline for MITS), before being grown in LB broth statically at 37 °C for 16 h. Cultures of individual WITS or MITS were pooled (as eight WITS or eight MITS), diluted in sterile PBS (Sigma–Aldrich) and injected iv into a lateral tail vein. Ampicillin sodium salt and ciprofloxacin hydrochloride powders (Sigma–Aldrich) were dissolved in endotoxin-free water (Sigma–Aldrich) and administered by ip injection (in 0.2 mL volume) at 12 h intervals for 4 days. The maximum recommended dosage for veterinary treatment of small rodents was used (150 mg/kg/dose for ampicillin treatment and 20 mg/kg/dose in the case of ciprofloxacin treatment).^31^^,^^32^

We chose an inoculum dose of 10^3^ cfu for infections with WITS. This allowed measurement of fluctuations in the heterogeneity of WITS subpopulations without stochastic loss of any subpopulation in livers and spleens and only moderate stochastic loss of subpopulations in MLNs at 72 h after iv infection.^12^ The MITS infection dose was set at 10^6^ cfu in order to achieve on day 3 (before the start of antimicrobial treatment) viable bacterial counts in the tissues at levels similar to those of the WITS.^33^

Groups of mice (Table S2) were killed before the start of the antimicrobial treatments (3 days post-infection) and every 24 h during the 4 days of treatment, and at two further timepoints after cessation of drugs (1 and 2 days, or 7 and 13 days after cessation of antimicrobials for WITS and MITS infections, respectively).

### Ethics

All animal experiments were performed in accordance with good animal practice as defined by the relevant international (Directive of the European Parliament and of the Council on the Protection of Animals Used for Scientific Purposes, Brussels 543/5) and local (University of Cambridge) animal welfare guidelines. This research has been regulated under the Animals (Scientific Procedures) Act 1986 Amendment Regulations 2012 following ethical review by the University of Cambridge Animal Welfare and Ethical Review Body (AWERB).

### Enumeration and recovery of viable Salmonella in organs

Blood was collected from a tail artery (approximately 0.4 mL of blood per animal) in heparin-coated tubes. Mice were killed by cervical dislocation and spleens, livers and MLNs were removed and individually homogenized in molecular-grade sterile water (5 mL for livers and spleens, 2 mL for MLNs) using a Stomacher 80 (Seward). Homogenates were plated onto LB agar. If required, serial 10-fold dilutions in PBS were used to enumerate viable bacteria. The cfu for the blood were calculated assuming a total circulating volume of 2 mL.

All the colonies obtained from each organ/blood after overnight incubation at 37 °C were harvested and resuspended in 2 mL of sterile PBS in the case of livers and spleens, or in 1 mL of sterile PBS for blood and MLNs samples. Recovered bacterial suspensions were thoroughly mixed, and aliquots were stored at −80 °C prior to genomic DNA extraction.

### Determination of ITS proportions in bacterial samples by amplicon sequencing

We determined ITS proportions within samples using a sequencing-based approach adapting the 16S library preparation guide from Illumina.^34^ Briefly, genomic DNA was purified from aliquots of the bacteria recovered from the agar plates (∼5 × 10^9^ cfu) using a DNeasy Blood and Tissue kit (Qiagen). 25 ng DNA from each sample was used as template for a first PCR to amplify 113 bp regions containing the unique nucleotide signatures from each sample (composed of a mix of the various DNA tags differentiating the various ITS). DNA was purified using AMPure beads (Beckman Coulter) and indexed (using a Nextera XT Index kit; Illumina) by a second PCR to enable the sequencing (and the multiplexing) by Illumina technology. For each sample, 25 ng of DNA was pooled, and the library was assayed and quality checked by NEBNext Library Quant Kit for Illumina (New England Biolabs). Finally, 20 pM of library (denatured with NaOH) was spiked with 5% PhiX control (Illumina) and sequenced using a MiSeq (Illumina). Automated image analysis, base-calling, data quality assessment and de-multiplexing were performed by the MiSeq instrument. The number of reads containing the specific tags was determined using an R-based script and this information was subsequently used to establish the presence, absence and relative abundance of each tagged strain within each sample.

### Statistical analysis and data processing

Variations in bacterial loads per organ were analysed using a linear model, with log-transformed cfu as a response variable, and strain, antimicrobial, organ, day and day^2^ (to account for non-linear dynamics) as explanatory variables, using the lm function in R version 3.3.^35^ We used analysis of variance (ANOVA) and backward elimination of non-significant (*P *>* *0.05) terms to produce a minimal model. The analysis was repeated separately for each strain, for MLNs only, and without the MLNs (because of their clearly distinct dynamics). The ANOVA tables are reported in Appendix 2 (Tables S3–S12).

In order to detect systemic movement of bacteria between organs, we then compared the distributions of the eight tags (WITS or MITS) between pairs of organs within mice at each timepoint. This was performed by bootstrapped null distributions (under the assumption that the ITS populations in the pair of organs considered had been sampled from the same pool) using the geometric mean of each bootstrap sample as the statistic. Bias correction was applied as proposed by Davison and Hinkley,^36^ and adjusting the estimated *P* values using the Holm–Bonferroni method.^37^ A complete description of the algorithm and the adjusted *P* values obtained are reported in Appendix 3 (Tables S13 and S14). All hypothesis tests were performed at the 5% significance level (*P *≤* *0.05).

WITS or MITS analysis by amplicon sequencing was validated, and proportionality was determined, as well as the level of background noise (i.e. reads assigned to WITS or MITS in the absence of that DNA). Consequently, a ‘technical’ cut-off equal to 1240 reads (representing the background noise) was applied, and thus any value less than 1240 was reset to zero. A ‘biological’ cut-off was also set to 1 bacterium: if the product of a total bacterial load with the proportion of a WITS or MITS was <1, then the frequency of that WITS or MITS was reset to zero.

## Results

### Efficacy of ampicillin and ciprofloxacin on bacteria with fast and slow in vivo net growth rates

We injected different groups of mice with either the fast-growing WITS or the slow-growing MITS. The mice received either ampicillin or ciprofloxacin from day 3 of the infection for 4 days.

**Figure 1.**
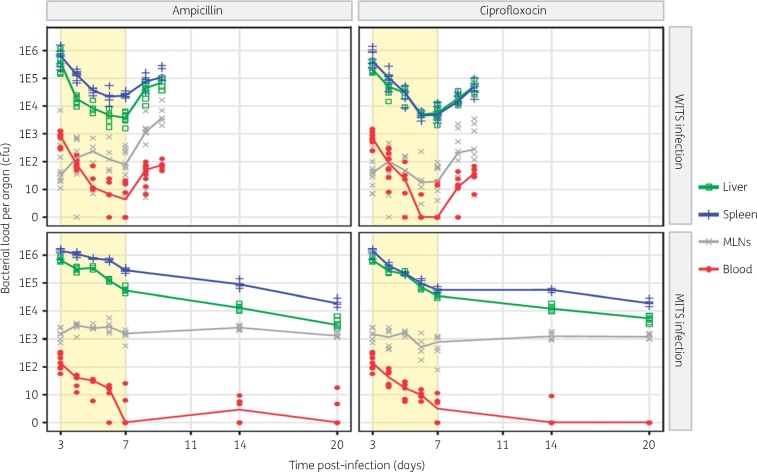
Bacterial loads in different organs at various timepoints post-infection. Different groups of C57BL/6 mice were infected iv with equal mixes of eight WITS to a total dose of ∼1000 cfu, or eight MITS for a total dose of ∼5 × 10^5^ cfu. Each symbol represents total cfu count from an organ of a single mouse. Solid lines join the median of cfu values for each organ. Light yellow background indicates the phase of antimicrobial treatment (ip for 4 days every 12 h). This figure appears in colour in the online version of *JAC* and in black and white in the printed version of *JAC*.

Figure 1 (top panels) shows a biphasic effect of both antimicrobials in the spleen and liver. The numbers of viable WITS decreased by approximately 99% in the first 3 days of treatment [days 3–6 post-infection (p.i.)] with no further significant reductions being observed after two more doses of either antimicrobial (i.e. between days 6 and 7 p.i.). This was confirmed statistically by a significant and positive quadratic effect of time in the ANOVA (Tables S4 and S7). Ampicillin showed a more marked effect on the overall reduction in viable bacterial numbers in the liver as compared with the spleen, while the effect of treatment with ciprofloxacin was similar in both organs (Table S7). The hepatic viable bacterial loads were similar in the livers at the end of treatment with either antimicrobial (*P *=* *0.17 by Mann–Whitney test at day 7 p.i.), whereas the efficacy of ampicillin was less than that of ciprofloxacin treatment in the spleen (*P *<* *1 × 10^−4^ by Mann–Whitney test at day 7 p.i.; Table S7). Bacteraemia was reduced by both antimicrobial treatments, with only a minority (6/20) of mice having detectable numbers of WITS (<10 cfu/mL) in the blood by day 7 p.i.. Treatment with ampicillin or ciprofloxacin did not reduce the viable numbers of WITS bacteria in MLNs (no effect of time, *P *=* *0.4, Table S6). However, cessation of the antimicrobial treatment resulted in the relapse of bacterial growth at similar rates in all the organs (3-fold increase per day), including MLNs, and the resurgence of bacteraemia (Table S10).

We then proceeded to study the effect of antimicrobial treatment on an infection with slow-growing MITS (Figure 1, bottom panels). In the infection with the attenuated strain both ampicillin and ciprofloxacin induced a lower and steady reduction in viable bacterial numbers compared with what was observed in the infection with WT bacteria in livers and spleens (approximately 90% in infection with MITS as compared with approximately 99% in the infection with WITS), with ciprofloxacin again having a stronger effect than ampicillin in the spleen (*P *= 0.01, Table S8). Bacteraemia was gradually reduced to below detectable limits in the majority (7/12) of animals by the end of the treatment. The viable numbers of MITS in MLNs remained unaffected by the treatment with either antimicrobial (removing time from the model had no significant effect on the ANOVA, Appendix 2). After the cessation of treatment, there was no evidence of relapse. MITS loads continued to slowly decrease in livers and spleens, bacteraemia remained undetectable or at a low level, while viable bacterial loads persisted in the MLNs at constant levels (Table S12).

### MLNs have different WITS and MITS population structures from those of the spleens, livers and blood

**Figure 2.**
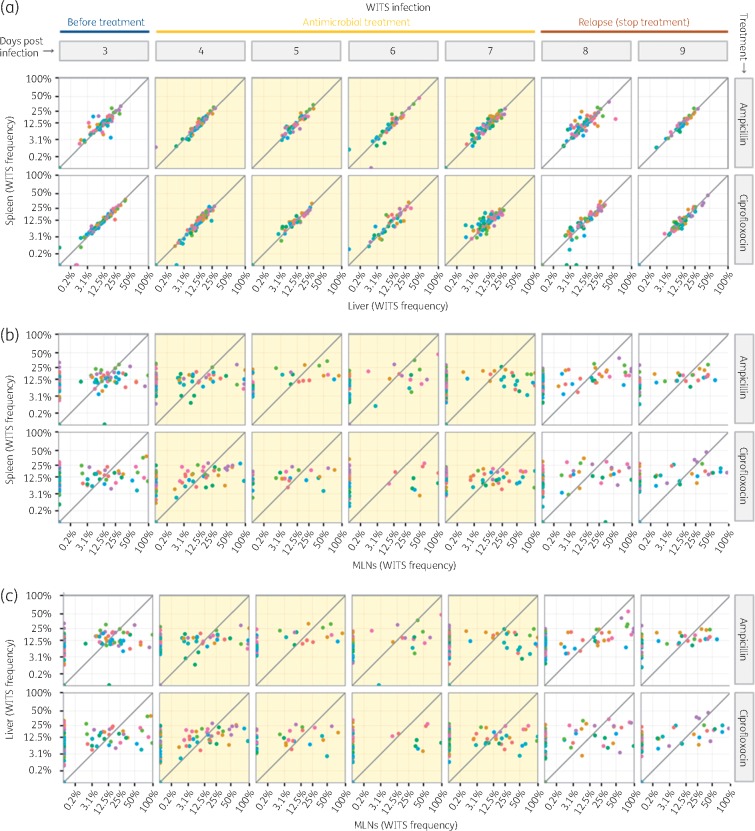
Pairwise distribution of the eight WITS in different organs for each mouse: (a) spleen versus liver; (b) spleen versus MLNs; and (c) liver versus MLNs. Each dot shows the frequency of a single WITS coded by colour. Each panel shows data from all the mice treated with a given antimicrobial and euthanized on a given day. Light yellow background indicates the time of antimicrobial treatment. This figure appears in colour in the online version of *JAC* and in black and white in the printed version of *JAC*.

We showed that antimicrobial treatment fails to reduce viable bacterial numbers in MLNs in contrast with the reduction in bacterial loads seen in the spleen, liver and blood. However, in infections with WITS a rapid resurgence of bacterial growth was seen in all organs including MLNs. This could be due to the growth of WITS from within MLNs following the interruption of antimicrobial treatment, or could be due to dissemination into the MLNs of bacterial populations growing in the spleen and liver. To test which of these scenarios was the most plausible, we compared the relative proportions of each of the WITS (population structure) in the spleen, liver and MLNs of each mouse at each timepoint, before, during and after antimicrobial treatment (Figure 2). We found that the WITS population structure was similar in the spleen and liver of each mouse (Figure 2a) (visually indicated by the dots, representing WITS frequencies, falling close to or on the diagonal line in the graphs). This was confirmed by pairwise comparisons of WITS composition between organs by bootstrap analysis (Supplementary Material Appendix 3). Within each mouse, the WITS population structure in MLNs was different from that of the spleen (Figure 2b) and liver (Figure 2c), throughout the experiment (all bootstrapped *P *<* *0.01, Appendix 3). These differences extended to the post-treatment phase of relapse, indicating that the increase in WITS cfu numbers in the MLNs could not have been driven by an influx of bacteria from the spleen or liver. We also found that on day 3 p.i. (before the start of treatment), 4 p.i. (after 1 day of treatment), and 8 and 9 p.i. (the relapse phase) all the WITS that were present in the blood were also present in the spleen and liver, but not in the MLNs, suggesting lack of colonization of MLNs from the blood (Appendix 4, Figure S1).

**Figure 3.**
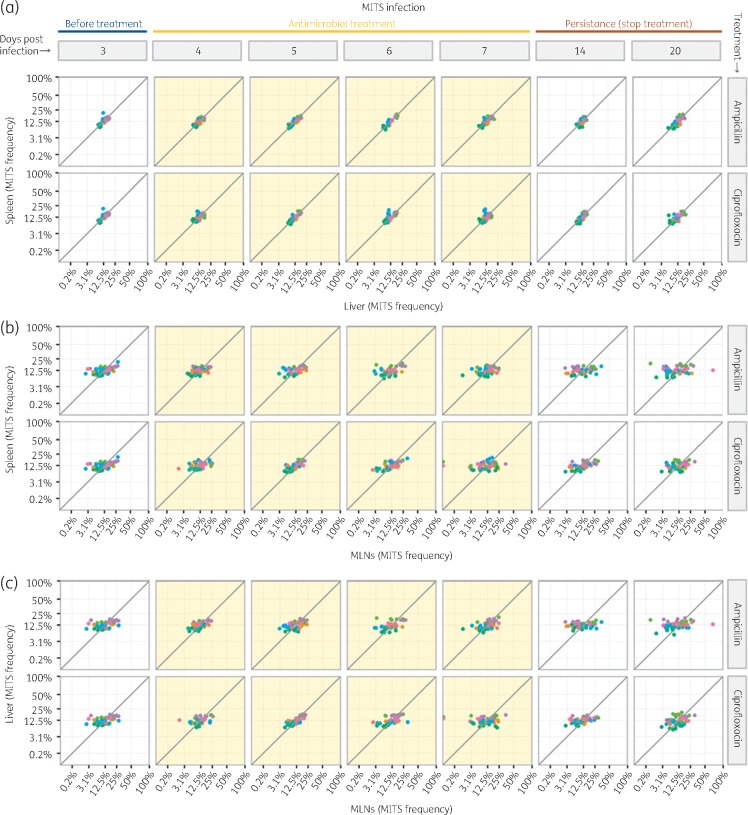
Pairwise distribution of the eight MITS in different organs for each mouse: (a) spleen versus liver; (b) spleen versus MLNs; and (c) liver versus MLNs. Each dot shows the frequency of a single MITS coded by colour. Each panel shows data from all the mice treated with a given antimicrobial and euthanized on a given day. Light yellow background indicates the time of antimicrobial treatment. This figure appears in colour in the online version of *JAC* and in black and white in the printed version of *JAC*.

Likewise, the population structure of the slow-growing MITS was indistinguishable between spleens and livers of each mouse (Figure 3a and Appendix 3), but different from that of the MLNs throughout the experiment (Figure 3b and c and Appendix 3). This indicates that the residual MITS population found in the MLNs after cessation of treatment does not significantly mix with that of the spleen and liver.

## Discussion

This study provides a direct *in vivo* comparison of the effects of treatment with two antimicrobials of clinical relevance on bacteria with fast or slow division rates. We showed that treatment with ampicillin or ciprofloxacin had a more marked *in vivo* effect on infections with fast-growing than slow-growing salmonellae.

We showed that the activity of ampicillin and ciprofloxacin is biphasic in infections with fast-growing bacteria but not with slow-growing ones. In fact, we found that the reduction in the numbers of bacteria that can be cultured from the spleen and liver homogenates occurs rapidly in the first 3 days of treatment with fast-growing WITS. No further reduction was observed on the fourth day following the last two doses of antimicrobials, leading to a situation where the bacteria are refractory to further antimicrobial treatment and persist in the tissues. The reasons for this biphasic effect of antimicrobial treatment are unclear. *Salmonella* is found in different cell types and also within multicellular pathological lesions that form at the sites of infections as a consequence of the onset and escalation of the innate immune response.^38–43^ These locations could represent privileged sites that are poorly accessible to antimicrobials where a fraction of the WITS bacterial load that escapes the initial rapid killing could persist. However, we did not observe the same biphasic effect of the treatment in mice infected with slow-growing MITS, where a constant decline in the number of bacteria that could be cultured was observed throughout the 4 days of treatment. This suggests that scenarios other than location are also likely to contribute to the dynamics of bacterial death and survival during antimicrobial treatment. For example, it is possible, and likely, that the selection of bacterial populations with low division rates and/or that are non-replicating are causal factors in post-treatment persistence.^44–49^ It is also possible that the heterogeneity in bacterial division rates or the percentage of non-replicating bacteria is higher in the overall initial (pre-antimicrobial treatment) WITS population as compared with the MITS, leading to the presence of fast-replicating WITS subpopulations easily killed by the antimicrobials and slower ones that may persist.

Our data show that neither antimicrobial treatment caused statistically significant changes in the viable bacterial numbers in MLNs. Although these have been reported as a site of *Salmonella* persistence,^47^^,^^48^^,^^50^^,^^51^ here we show that, surprisingly, MLNs are a site where no reductions in bacterial loads can be achieved by administration of ampicillin or ciprofloxacin. We currently do not have a simple mechanistic explanation for this phenomenon. It is possible that there is a fine balance between bacterial growth in between treatments in the MLNs and killing of bacteria by the antibiotics, leading to overall constant numbers of viable bacteria at the times of observation. It also possible that the majority of the bacteria in the MLNs enter a non-replicative state, with only a small proportion of bacteria growing rapidly in between treatments and being killed by the antibiotics. These growing populations would be responsible for the observed relapse of the infection after cessation of treatment. However, this hypothesis is in disagreement with: (i) the fact that the reported high initial percentage of non-replicating bacteria within MLNs is known to decrease as the infection progresses, with only approximately 10% of bacteria in the MLNs not having replicated (and therefore potentially refractory to antimicrobials) by 24 h after inoculation;^45^ and (ii) the observation that the heterogeneity of tagged strains within the MLNs does not increase during the treatment phase.

We found that the relapse of bacterial growth in the MLNs was not driven by migration of *Salmonella* from other body sites, as the compositions of WITS and MITS in the MLNs remained distinct from those of bacteria in livers and spleens. Overall this indicates that MLNs are a compartmentalized privileged site for *Salmonella* where the bacterial load becomes completely refractory to reductions via antimicrobial treatment and can resume growth or persist after cessation of therapy.

This study indicates complex *in vivo* scenarios. The mechanisms that underpin bacterial location, division and interaction with host cells *in vivo* under antimicrobial pressure within the complexity of a whole mammalian organism remain unclear. This lack of knowledge currently hampers our ability to design *in vivo* approaches to target those bacteria that are poorly susceptible to treatment.

## Supplementary Material

Supplementary Data
