# Supplementary material for: Within-host spatiotemporal dynamics of systemic Salmonella infection during and after antimicrobial treatment
Source: J Antimicrob Chemother. 2017 Aug 23;72(12):3390–7. doi: 10.1093/jac/dkx294 (PMC5890750; doi:10.1093/jac/dkx294)
Supplement: Supplementary Data [file supplementary_data_dkx294.docx]

**Supplementary data**

**Within-host spatiotemporal dynamics of systemic *Salmonella* infection during and after antimicrobial treatment**

O. Rossi, R. Dybowski, D.J. Maskell, A.J. Grant, O. Restif, P. Mastroeni

University of Cambridge, Department of Veterinary Medicine, Cambridge, United Kingdom

# Appendix 1. MUTANTS GENERATION AND CHARACTERIZATION

## Mutants generation and characterization

*Salmonella enterica* serovar Typhimurium SL1344,[^1^](#_ENREF_1) a virulent strain with an i.v. LD_50_ < 10 cfu for innately susceptible mice, was used as the parent strain. Standard methods and reagents were used for molecular cloning;[^2^](#_ENREF_2) all primers were purchased from Sigma Aldrich and are listed in Table S1.

The tetracycline resistance cassette *tetRA* was used to replace the *aroC* gene, and was amplified from a SL1344 Δ*htrA*::*tetRA* mutant[^3^](#_ENREF_3) using primers aroC-F/aroC-R. DNA fragments containing individual 40 bp signature tags and a kanamycin resistance cassette were inserted between the *malX‑Y* pseudogenes, and were amplified from individually tagged Wild-type Isogenic Tagged Strains (WITS) of *S.* Typhimurium JH3016, a Green Fluorescent Protein expressing derivative of wild-type virulent *S.* Typhimurium SL1344[^4^](#_ENREF_4) using primers ITS-F and ITS-R (amplifying an external region common to all the WITS). Approximately 1 μg of each linear PCR product was used for integration onto the chromosome of recombination-prone *Salmonella* recipient cells using a modification of the lambda red method [^5^](#_ENREF_5), as previously detailed. [^4^](#_ENREF_4) Transformants were selected by plating onto selective media (tetracycline 20 μg/mL for Δ*aroC,* kanamycin 50 μg/mL for WITS, a combination of the two for Δ*aroC* MITS), and verified by specific PCRs (performed using aroC-ext-R/Tet-Cont-F to confirm the *aroC* mutation, and primer “common” together a primer designed to the respective unique tag - tag1, tag 2, tag 11, tag 13, tag17, tag 19, tag 20, tag 21- to confirm isogenic tagging, respectively). Constructs were further confirmed by sequencing the DNA fragment modified (amplified by primers aroC-ext-F/aroC-ext-R and ITS-F/ITS-R respectively - Bioscience Cambridge, UK). Loss of the pBADλred helper plasmid (containing ampicillin resistance marker) was obtained by consecutive growth of positive transformants in liquid LB media without ampicillin selection for the plasmid, before plating an appropriate dilution (10^-6^) onto LB plates and using MAST ID intralactam circles (MAST Diagnostics, UK) to screen for the absence of beta-lactamase activity in bacterial colonies. The final results of the molecular cloning were a set of eight fast-growing wild-type isogenic tagged strains (WITS) and a set of eight slow-growing Δ*aroC* Mutant Isogenic Tagged Strains (MITS).[^1^](#_ENREF_1)^,^ [^3^](#_ENREF_3) Each of the eight WITS or MITS within a set contains a different 40 bp DNA signature tag in the same non-coding region of the chromosome, that enables the distinction and quantification of each tagged strain (subpopulation) within the set in a mixed sample.[^4^](#_ENREF_4)

All the Isogenic Tagged Strains composing each set were characterized and resulted to have the same Minimal Inhibitory Concentration to ampicillin and ciprofloxacin (performed using micro-well method),[^6^](#_ENREF_6) as well as *in vitro* (cfu/mL after static growth for 16 hours in 10 mL of LB liquid culture at 37°C) and *in vivo* fitness (assessed by determining cfu/organ in spleens and livers three days after i.v. infection of C57BL/6 mice with 10^3^ cfu and 10^5^ cfu for WITS and MITS in sterile PBS, respectively).

**Table S1.** List of primers used in this study

| **Primer name** | **Sequence (5’→3’)** |
| --- | --- |
| ITS-F | GAGCGGCTATCGTTTGACTC |
| ITS-R | AAACGATCGGCGACATAATC |
| aroC-ext-F | ccgttcacctggctggagttt |
| aroC-PCR-F | acatttcaatatttataaaga |
| aroC-F | acatttcaatatttataaagattaaaacacgcaaacgacaacaacgataacggagccgtgttgacagcttatcatcgata |
| aroC-R | cgccaggctggcgctactgacaaaccatgccagcagcgcaatcgcggtttttttcatttctcaggtcgaggtggcccggc |
| aroC-PCR-R | cgccaggctggcgctactgac |
| aroC-ext-R | atgatgcatccgttggcaaag |
| Tet-cont-F | atcgtcgccgcacttatgact |
| Kan-cont-F | gcctgttgaacaagtctggaa |
| tag 1 | acgacaccactccacaccta |
| tag 2 | acccgcaataccaacaactc |
| tag 11 | atcccacacactcgatctca |
| tag 13 | gctaaagacacccctcactca |
| tag 17 | tcaccagcccaccccctca |
| tag 19 | gcactatccagccccataac |
| tag 20 | acctaactataccgccatcc |
| tag 21 | acaaccaccgatcactctcc |
| common tag | cacggaaaacatcgtgagtc |
| MiSeq-F | TCGTCGGCAGCGTCAGATGTGTATAAGAGACAGGGTTGGCCTGATGAATACCG |
| MiSeq-R | GTCTCGTGGGCTCGGAGATGTGTATAAGAGACAGcttgtgcaatgtaacatcagaga |

**Table S2.** **Experimental groups.** Number of mice sacrificed at various time-point. Note that in pilot experiments further five mice per time point (day 3, 7 and 8 post infection) were sacrificed for determining bacterial loads and ITS distribution in the organs for the 10^4^ and 10^2^ cfu infection doses. Ampicillin and ciprofloxacin treatments of Δ*aroC* infections were conducted in parallel to minimize the amount of mice, thus mice at day three post infection (before the start of antimicrobial treatment) refer to same animals.

| **Infection – treatment** | **Days post-infection** | | | | | | | | |
| --- | --- | --- | --- | --- | --- | --- | --- | --- | --- |
|  | **3** | **4** | **5** | **6** | **7** | **8** | **9** | **14** | **20** |
| WITS – Ampicillin | 10 | 10 | 5 | 5 | 10 | 10 | 5 | - | - |
| WITS – Ciprofloxacin | 10 | 10 | 5 | 5 | 10 | 8 | 7 | - | - |
| MITS - Ampicillin | 7 | 6 | 5 | 5 | 6 | - | - | 6 | 6 |
| MITS - Ciprofloxacin |  | 6 | 5 | 5 | 6 | - | - | 6 | 6 |

# Appendix 2. ANALYSIS OF BACTERIAL LOADS

To compare bacterial loads within and between organs across time points analysis of variance (ANOVA) of trend for total bacterial load during antimicrobial treatments (days three to seven post-infection) and during the post-treatment phase (day seven onwards) was performed across the time points of interest, using a linear model for log_10_ cfu, under the assumption that the residuals about the regression line were normally distributed with similar variance over time. The assumptions of linearity, homoscedasticity and normality were checked by the inspection of diagnostic plots. The possibility of interactions was taken into account by including multiplicative interaction terms in the models. All hypothesis tests were performed at the 5 % significance level (*p* **≤** 0.05).

## Response to antimicrobial treatment (days 3-7 post infection)

#### **Table S3.** All groups togheter, complete model.

Df Sum Sq Mean Sq F value Pr(>F)
Strain 1 116.42 116.42 872.0778 < 2.2e-16 ***
Drug 1 6.66 6.66 49.9205 7.754e-12 ***
Organ 2 648.63 324.31 2429.4388 < 2.2e-16 ***
Day.t 1 61.33 61.33 459.4430 < 2.2e-16 ***
I(Day.t^2) 1 0.58 0.58 4.3568 0.0375305 *
Strain:Drug 1 0.25 0.25 1.8712 0.1721509
Strain:Organ 2 5.10 2.55 19.0912 1.263e-08 ***
Drug:Organ 2 4.51 2.26 16.8960 9.372e-08 ***
Strain:Day.t 1 2.71 2.71 20.3336 8.691e-06 ***
Strain:I(Day.t^2) 1 2.50 2.50 18.6907 1.969e-05 ***
Drug:Day.t 1 2.00 2.00 15.0121 0.0001259 ***
Drug:I(Day.t^2) 1 0.00 0.00 0.0120 0.9128586
Organ:Day.t 2 22.11 11.06 82.8153 < 2.2e-16 ***
Organ:I(Day.t^2) 2 3.87 1.93 14.4926 8.621e-07 ***
Strain:Drug:Organ 2 0.85 0.42 3.1663 0.0432779 *
Strain:Drug:Day.t 1 0.11 0.11 0.8102 0.3686249
Strain:Drug:I(Day.t^2) 1 0.56 0.56 4.1619 0.0420377 *
Strain:Organ:Day.t 2 1.56 0.78 5.8617 0.0031116 **
Strain:Organ:I(Day.t^2) 2 1.14 0.57 4.2693 0.0146717 *
Drug:Organ:Day.t 2 1.27 0.64 4.7662 0.0090307 **
Drug:Organ:I(Day.t^2) 2 0.46 0.23 1.7255 0.1794812
Strain:Drug:Organ:Day.t 2 0.17 0.08 0.6187 0.5391822
Strain:Drug:Organ:I(Day.t^2) 2 0.13 0.07 0.4942 0.6104393
Residuals 378 50.46 0.13
---
Signif. codes: 0 '***' 0.001 '**' 0.01 '*' 0.05 '.' 0.1 ' ' 1

#### **Table S4**. All groups, minimal model after stepwise elimination of non-significant interaction terms.

Df Sum Sq Mean Sq F value Pr(>F)
Strain 1 116.42 116.42 868.3508 < 2.2e-16 ***
Drug 1 6.66 6.66 49.7072 8.253e-12 ***
Organ 2 648.63 324.31 2419.0561 < 2.2e-16 ***
Day.t 1 61.33 61.33 457.4795 < 2.2e-16 ***
I(Day.t^2) 1 0.58 0.58 4.3382 0.0379225 *
Strain:Drug 1 0.25 0.25 1.8632 0.1730492
Strain:Organ 2 5.10 2.55 19.0096 1.334e-08 ***
Drug:Organ 2 4.51 2.26 16.8238 9.860e-08 ***
Strain:Day.t 1 2.71 2.71 20.2467 9.015e-06 ***
Strain:I(Day.t^2) 1 2.50 2.50 18.6108 2.038e-05 ***
Drug:Day.t 1 2.00 2.00 14.9479 0.0001296 ***
Organ:Day.t 2 22.11 11.06 82.4614 < 2.2e-16 ***
Organ:I(Day.t^2) 2 3.87 1.93 14.4307 9.027e-07 ***
Strain:Drug:Organ 2 0.85 0.42 3.1528 0.0438315 *
Strain:Organ:Day.t 2 1.56 0.78 5.8366 0.0031817 **
Strain:Organ:I(Day.t^2) 2 1.14 0.57 4.2511 0.0149197 *
Drug:Organ:Day.t 2 1.27 0.64 4.7459 0.0091996 **
Residuals 387 51.88 0.13

Analysis of Variance Table
Res.Df RSS Df Sum of Sq F Pr(>F)
1 387 51.883
2 378 50.460 9 1.4232 1.1846 0.3034

#### **Table S5.** MLNs only, complete model.

Df Sum Sq Mean Sq F value Pr(>F)
Strain 1 65.023 65.023 199.0223 < 2.2e-16 ***
Drug 1 6.062 6.062 18.5533 3.294e-05 ***
Day.t 1 0.471 0.471 1.4412 0.23220
I(Day.t^2) 1 1.351 1.351 4.1362 0.04407 *
Strain:Drug 1 0.171 0.171 0.5224 0.47114
Strain:Day.t 1 0.002 0.002 0.0063 0.93706
Strain:I(Day.t^2) 1 0.008 0.008 0.0247 0.87543
Drug:Day.t 1 0.961 0.961 2.9400 0.08887 .
Drug:I(Day.t^2) 1 0.131 0.131 0.4011 0.52767
Strain:Drug:Day.t 1 0.000 0.000 0.0000 0.99508
Strain:Drug:I(Day.t^2) 1 0.027 0.027 0.0825 0.77442
Residuals 126 41.166 0.327

#### **Table S6.** MLNs only, minimal model.

Df Sum Sq Mean Sq F value Pr(>F)
Strain 1 65.023 65.023 198.208 < 2.2e-16 ***
Drug 1 6.062 6.062 18.477 3.27e-05 ***
Residuals 135 44.287 0.328
---
Signif. codes: 0 '***' 0.001 '**' 0.01 '*' 0.05 '.' 0.1 ' ' 1

Analysis of Variance Table

Model 1: Y ~ Strain * Drug * (Day.t + I(Day.t^2))
Model 2: Y ~ Strain + Drug + I(Day.t^2)
Model 3: Y ~ Strain + Drug
 Res.Df RSS Df Sum of Sq F Pr(>F)
1 126 41.166
2 134 43.348 -8 -2.18251 0.8350 0.57345
3 135 44.287 -1 -0.93904 2.8742 0.09248 .

#### **Table S7.** WITS infections, Liver and Spleen only, complete model.

Analysis of Variance Table

Df Sum Sq Mean Sq F value Pr(>F)
Drug 1 0.246 0.246 4.4148 0.037324 *
Organ 1 6.405 6.405 115.1684 < 2.2e-16 ***
Day.t 1 68.839 68.839 1237.7854 < 2.2e-16 ***
I(Day.t^2) 1 6.908 6.908 124.2043 < 2.2e-16 ***
Drug:Organ 1 2.407 2.407 43.2734 7.697e-10 ***
Drug:Day.t 1 0.318 0.318 5.7155 0.018073 *
Drug:I(Day.t^2) 1 0.589 0.589 10.5901 0.001409 **
Organ:Day.t 1 0.000 0.000 0.0023 0.961415
Organ:I(Day.t^2) 1 0.101 0.101 1.8131 0.180195
Drug:Organ:Day.t 1 1.118 1.118 20.1074 1.459e-05 ***
Drug:Organ:I(Day.t^2) 1 0.236 0.236 4.2442 0.041136 *
Residuals 148 8.231 0.056

Coefficients:
 Estimate Std. Error t value
(Intercept) 5.47504 0.07104 77.066
DrugCiprofloxacin -0.13993 0.10047 -1.393
OrganSpleen 0.30792 0.10047 3.065
Day.t -1.27136 0.09520 -13.355
I(Day.t^2) 0.20121 0.02260 8.903
DrugCiprofloxacin:OrganSpleen 0.08167 0.14209 0.575
DrugCiprofloxacin:Day.t 0.54001 0.13463 4.011
DrugCiprofloxacin:I(Day.t^2) -0.12011 0.03196 -3.758
OrganSpleen:Day.t 0.42126 0.13463 3.129
OrganSpleen:I(Day.t^2) -0.07699 0.03196 -2.409
DrugCiprofloxacin:OrganSpleen:Day.t -0.59699 0.19040 -3.135
DrugCiprofloxacin:OrganSpleen:I(Day.t^2) 0.09312 0.04520 2.060
 Pr(>|t|)
(Intercept) < 2e-16 ***
DrugCiprofloxacin 0.165786
OrganSpleen 0.002590 **
Day.t < 2e-16 ***
I(Day.t^2) 1.80e-15 ***
DrugCiprofloxacin:OrganSpleen 0.566295
DrugCiprofloxacin:Day.t 9.56e-05 ***
DrugCiprofloxacin:I(Day.t^2) 0.000246 ***
OrganSpleen:Day.t 0.002113 **
OrganSpleen:I(Day.t^2) 0.017231 *
DrugCiprofloxacin:OrganSpleen:Day.t 0.002070 **
DrugCiprofloxacin:OrganSpleen:I(Day.t^2) 0.041136 *

Residual standard error: 0.2358 on 148 degrees of freedom
Multiple R-squared: 0.9137, Adjusted R-squared: 0.9073
F-statistic: 142.5 on 11 and 148 DF, p-value: < 2.2e-16

#### **Table S8**. MITS infection, Liver and Spleen only, complete model.

Df Sum Sq Mean Sq F value Pr(>F)
Drug 1 2.5906 2.5906 253.2877 < 2.2e-16 ***
Organ 1 3.4620 3.4620 338.4857 < 2.2e-16 ***
Day.t 1 17.9276 17.9276 1752.7903 < 2.2e-16 ***
I(Day.t^2) 1 0.0385 0.0385 3.7661 0.0550100 .
Drug:Organ 1 0.7951 0.7951 77.7384 2.924e-14 ***
Drug:Day.t 1 0.9132 0.9132 89.2885 1.144e-15 ***
Drug:I(Day.t^2) 1 0.1513 0.1513 14.7919 0.0002072 ***
Organ:Day.t 1 0.1248 0.1248 12.1970 0.0007041 ***
Organ:I(Day.t^2) 1 0.0377 0.0377 3.6862 0.0576055 .
Drug:Organ:Day.t 1 0.2116 0.2116 20.6857 1.469e-05 ***
Drug:Organ:I(Day.t^2) 1 0.0439 0.0439 4.2933 0.0407360 *
Residuals 104 1.0637 0.0102

Coefficients:
 Estimate Std. Error t value
(Intercept) 5.801857 0.036365 159.544
DrugCiprofloxacin 0.020139 0.051428 0.392
OrganSpleen 0.313339 0.051428 6.093
Day.t -0.133805 0.046028 -2.907
I(Day.t^2) -0.031457 0.011166 -2.817
DrugCiprofloxacin:OrganSpleen -0.029719 0.072730 -0.409
DrugCiprofloxacin:Day.t -0.141049 0.065094 -2.167
DrugCiprofloxacin:I(Day.t^2) 0.019808 0.015791 1.254
OrganSpleen:Day.t 0.109429 0.065094 1.681
OrganSpleen:I(Day.t^2) -0.001698 0.015791 -0.108
DrugCiprofloxacin:OrganSpleen:Day.t -0.299441 0.092056 -3.253
DrugCiprofloxacin:OrganSpleen:I(Day.t^2) 0.046271 0.022331 2.072
 Pr(>|t|)
(Intercept) < 2e-16 ***
DrugCiprofloxacin 0.69616
OrganSpleen 1.9e-08 ***
Day.t 0.00446 **
I(Day.t^2) 0.00580 **
DrugCiprofloxacin:OrganSpleen 0.68366
DrugCiprofloxacin:Day.t 0.03253 *
DrugCiprofloxacin:I(Day.t^2) 0.21251
OrganSpleen:Day.t 0.09574 .
OrganSpleen:I(Day.t^2) 0.91456
DrugCiprofloxacin:OrganSpleen:Day.t 0.00154 **
DrugCiprofloxacin:OrganSpleen:I(Day.t^2) 0.04074 *

Residual standard error: 0.1011 on 104 degrees of freedom
Multiple R-squared: 0.9611, Adjusted R-squared: 0.957
F-statistic: 233.7 on 11 and 104 DF, p-value: < 2.2e-16

## Post-treatment phase (day 7 post infection onwards)

After cessation of treatment, we only have two time points (days 8 and 9 for the wild-type; days 14 and 20 for the mutant), so we did not include quadratic terms in the models.

#### **Table S9.** WITS infection, complete model.

Df Sum Sq Mean Sq F value Pr(>F)
Drug 1 6.353 6.353 28.3942 3.929e-07 ***
Organ 2 140.535 70.268 314.0640 < 2.2e-16 ***
Day.r 1 31.627 31.627 141.3592 < 2.2e-16 ***
Drug:Organ 2 2.291 1.146 5.1207 0.007157 **
Drug:Day.r 1 0.271 0.271 1.2096 0.273328
Organ:Day.r 2 1.681 0.841 3.7571 0.025774 *
Drug:Organ:Day.r 2 0.471 0.235 1.0525 0.351841
Residuals 138 30.876 0.224

#### **Table S10.** WITS infections, minimal model.

Df Sum Sq Mean Sq F value Pr(>F)
Drug 1 6.353 6.353 28.3309 3.934e-07 ***
Organ 2 140.535 70.268 313.3647 < 2.2e-16 ***
Day.r 1 31.627 31.627 141.0444 < 2.2e-16 ***
Drug:Organ 2 2.291 1.146 5.1093 0.007207 **
Organ:Day.r 2 1.681 0.841 3.7487 0.025927 *
Residuals 141 31.617 0.224

Coefficients:
 Estimate Std. Error t value Pr(>|t|)
(Intercept) 3.74389 0.11856 31.579 < 2e-16 ***
DrugCiprofloxacin -0.10179 0.13407 -0.759 0.448972
OrganSpleen 0.60225 0.16766 3.592 0.000452 ***
OrganMLN -1.82278 0.16766 -10.872 < 2e-16 ***
Day.r 0.49448 0.07507 6.587 8.27e-10 ***
DrugCiprofloxacin:OrganSpleen -0.50411 0.18960 -2.659 0.008750 **
DrugCiprofloxacin:OrganMLN -0.54883 0.18960 -2.895 0.004401 **
OrganSpleen:Day.r -0.11390 0.10616 -1.073 0.285159
OrganMLN:Day.r 0.17467 0.10616 1.645 0.102143

Residual standard error: 0.4735 on 141 degrees of freedom
Multiple R-squared: 0.8523, Adjusted R-squared: 0.8439
F-statistic: 101.7 on 8 and 141 DF, p-value: < 2.2e-16

**Table S11.** MITS infection, complete model.

Df Sum Sq Mean Sq F value Pr(>F)
Drug 1 1.029 1.0294 34.6188 5.851e-08 ***
Organ 2 50.170 25.0852 843.6273 < 2.2e-16 ***
Day.r 1 5.307 5.3072 178.4835 < 2.2e-16 ***
Drug:Organ 2 0.406 0.2032 6.8336 0.00168 **
Drug:Day.r 1 1.014 1.0145 34.1176 7.072e-08 ***
Organ:Day.r 2 5.084 2.5419 85.4844 < 2.2e-16 ***
Drug:Organ:Day.r 2 0.121 0.0606 2.0379 0.13591
Residuals 96 2.855 0.0297

#### **Table S12.** MITS infection, minimal model.

Df Sum Sq Mean Sq F value Pr(>F)
Drug 1 1.029 1.0294 33.9007 7.352e-08 ***
Organ 2 50.170 25.0852 826.1291 < 2.2e-16 ***
Day.r 1 5.307 5.3072 174.7815 < 2.2e-16 ***
Drug:Organ 2 0.406 0.2032 6.6919 0.001887 **
Drug:Day.r 1 1.014 1.0145 33.4099 8.871e-08 ***
Organ:Day.r 2 5.084 2.5419 83.7113 < 2.2e-16 ***
Residuals 98 2.976 0.0304

Coefficients:
 Estimate Std. Error t value Pr(>|t|)
(Intercept) 4.785234 0.058805 81.375 < 2e-16 ***
DrugCiprofloxacin -0.267233 0.071728 -3.726 0.000326 ***
OrganSpleen 0.658111 0.077656 8.475 2.44e-13 ***
OrganMLN -1.538290 0.077656 -19.809 < 2e-16 ***
Day.r -0.096124 0.006313 -15.227 < 2e-16 ***
DrugCiprofloxacin:OrganSpleen -0.280913 0.082144 -3.420 0.000915 ***
DrugCiprofloxacin:OrganMLN -0.232913 0.082144 -2.835 0.005560 **
DrugCiprofloxacin:Day.r 0.036488 0.006313 5.780 8.87e-08 ***
OrganSpleen:Day.r 0.015221 0.007731 1.969 0.051801 .
OrganMLN:Day.r 0.093236 0.007731 12.060 < 2e-16 ***

Residual standard error: 0.1743 on 98 degrees of freedom
Multiple R-squared: 0.9549, Adjusted R-squared: 0.9508
F-statistic: 230.6 on 9 and 98 DF, p-value: < 2.2e-16

# Appendix 3. COMPARISONS OF TAGS DISTRIBUTIONS BETWEEN ORGANS

To compare the WITS or MITS abundance between different organs of different mice from the same time-point, we compared the distributions of the eight WITS or MITS within a specific time point in a certain organ with the WITS or MITS frequencies resulting from observations made from the same mice but in different organs. This was done using bootstrapped null distributions.

Let X_t_ denote the matrix of WITS or MITS frequencies obtained from m mice with respect to a specified time point t and internal organ (e.g., liver). Let Y_t_ be a matrix of WITS or MITS frequencies resulting from observations made from the same m mice used for X_t_ but associated with a different organ (*e.g*., spleen). For comparing the distributions of the eight WITS or MITS between X_t_ and Y_t_, we used an approach based on bootstrap null distributions as follows. First, the WITS or MITS frequencies in the i-th row of X_t_, x_t,i_ were sorted in ascending order and then normalised by replacing the frequencies with their relative frequencies (relative to sum(x_t,i_)). We applied the same process to the i-th row, y_t,i_, of Y_t_, with the number of each WITS or MITS present replaced by its relative frequency. In order to reject the hypothesis that x_t,i_ and y_t,i_ originate from the same distribution, a bootstrap null distribution was set up by taking 1,000 bootstrap samples of size sum(y_t,i_) from x_t,i_ and using the geometric mean of each bootstrap sample as the statistic. The *P*-value of the geometric mean of y_t,i_ was estimated with respect to the simulated null distribution using the bias correction proposed by Davidson and Hinkley.[^11^](#_ENREF_11) This procedure was repeated for each (x_t,i_, y_t,i_) pair. In order to take into account that the use of multiple hypothesis tests inflates the false positive rate, the estimated *P*-values were adjusted using the Holm-Bonferonni method,[^12^](#_ENREF_12) and the minimum adjusted *P*- value was reported.

**Table S13.** *P*-values resulting from hypothesis tests for changes in WITS distributions using geometric mean and bootstrapped null (significant *P*-values in bold).

| **WITS infection** | | | |
| --- | --- | --- | --- |
| **Days post infection** |  | **Ciprofloxacin** | **Ampicillin** |
| Day 3 | Liver *vs* Spleen | 1 | 0.719 |
|  | Liver *vs* MLNs | **0.010** | **0.010** |
|  | Spleen *vs* MLNs | **0.010** | **0.010** |
| Day 4 | Liver *vs* Spleen | 0.130 | 0.859 |
|  | Liver *vs* MLNs | **0.009** | **0.009** |
|  | Spleen *vs* MLNs | **0.009** | **0.009** |
| Day 5 | Liver *vs* Spleen | 0.265 | 0.180 |
|  | Liver *vs* MLNs | **0.004** | **0.005** |
|  | Spleen *vs* MLNs | **0.004** | **0.005** |
| Day 6 | Liver *vs* Spleen | 0.215 | 0.390 |
|  | Liver *vs* MLNs | **0.005** | **0.005** |
|  | Spleen *vs* MLNs | **0.005** | **0.005** |
| Day 7 | Liver *vs* Spleen | **0.040** | 1 |
|  | Liver *vs* MLNs | **0.009** | **0.010** |
|  | Spleen *vs* MLNs | **0.009** | **0.010** |
| Day 8 | Liver *vs* Spleen | 0.280 | 0.100 |
|  | Liver *vs* MLNs | **0.008** | **0.010** |
|  | Spleen *vs* MLNs | **0.008** | **0.010** |
| Day 9 | Liver *vs* Spleen | 0.937 | 0.634 |
|  | Liver *vs* MLNs | **0.007** | **0.005** |
|  | Spleen *vs* MLNs | **0.007** | **0.005** |

**Table S14.** *P*-values resulting from hypothesis tests for changes in MITS distributions using geometric mean and bootstrapped null (significant *P*-values in bold).

| **MITS infection** | | | |
| --- | --- | --- | --- |
| **Days post infections** |  | **Ciprofloxacin** | **Ampicillin** |
| Day 3 | Liver *vs* Spleen | 0.797 | 0.797 |
|  | Liver *vs* MLNs | **0.007** | **0.007** |
|  | Spleen *vs* MLNs | **0.007** | **0.007** |
| Day 4 | Liver *vs* Spleen | 1 | 0.276 |
|  | Liver *vs* MLNs | **0.006** | **0.006** |
|  | Spleen *vs* MLNs | **0.006** | **0.006** |
| Day 5 | Liver *vs* Spleen | 1 | 0.415 |
|  | Liver *vs* MLNs | 0.175 | **0.005** |
|  | Spleen *vs* MLNs | 0.065 | **0.005** |
| Day 6 | Liver *vs* Spleen | 0.824 | 0.300 |
|  | Liver *vs* MLNs | **0.005** | **0.005** |
|  | Spleen *vs* MLNs | **0.005** | **0.005** |
| Day 7 | Liver *vs* Spleen | 1 | 1 |
|  | Liver *vs* MLNs | **0.006** | **0.006** |
|  | Spleen *vs* MLNs | **0.006** | **0.006** |
| Day 14 | Liver *vs* Spleen | 1 | 1 |
|  | Liver *vs* MLNs | **0.006** | **0.006** |
|  | Spleen *vs* MLNs | **0.006** | **0.006** |
| Day 20 | Liver *vs* Spleen | 0.216 | 0.498 |
|  | Liver *vs* MLNs | **0.006** | **0.006** |
|  | Spleen *vs* MLNs | **0.006** | **0.006** |

# Appendix 4. SUPPLEMENTARY DATA

**
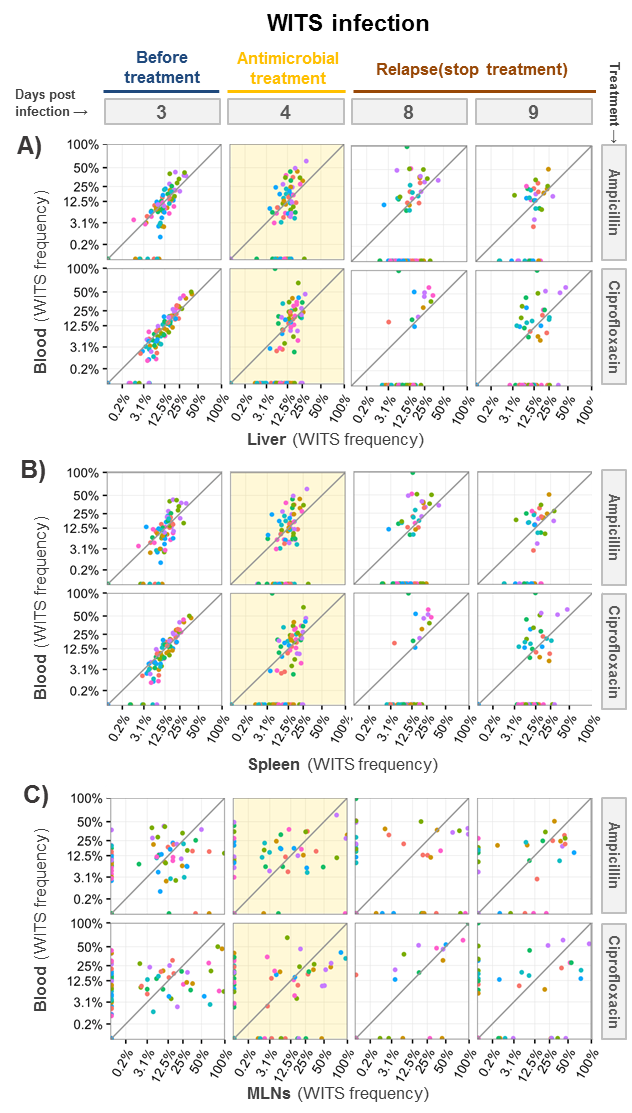
**

## Figure S1. Pairwise distribution of the eight WITS in the blood (y axis) and in other organs (x axis) for the each mouse (A. Blood *vs* Liver, B. Blood *vs* Spleen, C. Blood *vs* MLNs). Each dot shows the frequency of a single WITS coded by colour. Each panel shows data from all the mice treated with a given antimicrobial and sacrificed on a given day. Light yellow background indicates the time of antimicrobial treatment. This figure appears in colour in the online version of JAC and in black and white in the printed version of JAC.

## Reference list

1. Hoiseth SK, Stocker BA. Aromatic-dependent Salmonella typhimurium are non-virulent and effective as live vaccines. *Nature* 1981; **291**: 238-9.

2. Sambrook J, Russell DW. *Molecular cloning: a laboratory manual*. Cold Spring Harbor, NY: Cold Spring Harbor Laboratory, 2001.

3. Grant AJ, Oshota O, Chaudhuri RR *et al.* Genes Required for the Fitness of Salmonella enterica Serovar Typhimurium during Infection of Immunodeficient gp91-/- phox Mice. *Infect Immun* 2016; **84**: 989-97.

4. Grant AJ, Restif O, McKinley TJ *et al.* Modelling within-host spatiotemporal dynamics of invasive bacterial disease. *PLoS Biol* 2008; **6**: e74.

5. Datsenko KA, Wanner BL. One-step inactivation of chromosomal genes in Escherichia coli K-12 using PCR products. *Proc Natl Acad Sci U S A* 2000; **97**: 6640-5.

6. Wiegand I, Hilpert K, Hancock RE. Agar and broth dilution methods to determine the minimal inhibitory concentration (MIC) of antimicrobial substances. *Nat Protoc* 2008; **3**: 163-75.

7. Carpenter JW. *Exotic animal formulary*. Philadelphia, Pa. ; London: Saunders, 2012.

8. Hawk CT, Leary SL, Morris TH *et al.* *Formulary for laboratory animals*. Ames ; Oxford: Blackwell, 2005.

9. Grant AJ, Morgan FJ, McKinley TJ *et al.* Attenuated Salmonella Typhimurium lacking the pathogenicity island-2 type 3 secretion system grow to high bacterial numbers inside phagocytes in mice. *PLoS Pathog* 2012; **8**: e1003070.

10. Illumina. 16S Metagenomic sequencing library preparation. 2013; (Access Date Access 2013, date last accessed).

11. Davison AC, Hinkley DV. *Bootstrap Methods and their Application*. Cambridge: Cambridge University Press, 1997.

12. Holm S. A Simple Sequentially Rejective Multiple Test Procedure. *Scandinavian Journal of Statistics* 1979; **6**: 65-70.
